# Cough and Exhaled Nitric Oxide Levels: What Happens with Exercise?

**DOI:** 10.3389/fped.2013.00030

**Published:** 2013-10-24

**Authors:** Helen L. Petsky, Jennifer Anne Kynaston, Margaret McElrea, Catherine Turner, Alan Isles, Anne B. Chang

**Affiliations:** ^1^Queensland Children’s Medical Research Institute, Queensland University of Technology, Brisbane, QLD, Australia; ^2^Queensland Children’s Respiratory Centre, Royal Children’s Hospital, Brisbane, QLD, Australia; ^3^School of Nursing and Midwifery, The University of Queensland, Brisbane, QLD, Australia; ^4^General Paediatrics, Royal Children’s Hospital, Brisbane, QLD, Australia; ^5^Child Health Division, Menzies School of Health, Darwin, NT, Australia

**Keywords:** cough, pediatrics, exercise-induced broncho-constriction, atopy, FeNO

## Abstract

Cough associated with exertion is often used as a surrogate marker of asthma. However, to date there are no studies that have objectively measured cough in association with exercise in children. Our primary aim was to examine whether children with a pre-existing cough have an increase in cough frequency during and post-exercise. We hypothesized that children with any coughing illness will have an increase in cough frequency post-exercise regardless of the presence of exercise-induced broncho-constriction (EIB) or atopy. In addition, we hypothesized that Fractional exhaled nitric oxide (FeNO) levels decreases post-exercise regardless of the presence of EIB or atopy. Children with chronic cough and a control group without cough undertook an exercise challenge, FeNO measurements and a skin prick test, and wore a 24-h voice recorder to objectively measure cough frequency. The association between recorded cough frequency, exercise, atopy, and presence of EIB was tested. We also determined if the change in FeNO post exercise related to atopy or EIB. Of the 50 children recruited (35 with cough, 15 control), 7 had EIB. Children with cough had a significant increase in cough counts (median 7.0, inter-quartile ranges, 0.5, 24.5) compared to controls (2.0, IQR 0, 5.0, *p* = 0.028) post-exercise. Presence of atopy or EIB did not influence cough frequency. FeNO level was significantly lower post-exercise in both groups but the change was not influenced by atopy or EIB. Cough post-exertion is likely a generic response in children with a current cough. FeNO level decreases post-exercise irrespective of the presence of atopy or EIB. A larger study is necessary confirm or refute our findings.

## Introduction

Cough is one of the most common symptoms presenting to doctors and when present in children, is associated with impaired quality of life and burden to parents (1). Cough associated with exercise is often considered a symptom of asthma. (2, 3) However, some clinicians have observed that exercise can exacerbate cough in any child with a pre-existing cough, irrespective of diagnosis. Yet, in adult studies on cough sensitivity to fog (which is dependent on minute volume), desensitization of the cough reflex occurs post-exercise (4). Increased cough is usually associated with increased cough reflex sensitivity. (5) Thus, there is controversy on the relationship between cough and exercise and there are currently no published data that have measured cough frequency objectively pre- and post-exercise in children. Indeed there is little published research on cough and exercise in either adults or children (6).

Fractional exhaled nitric oxide (FeNO), is an eosinophilic inflammatory marker measured by a non-invasive test. It is elevated in steroid-naïve children with atopy and asthma. Some have advocated its use to diagnose and/or monitor asthma (7). There are no reported studies examining FeNO in children with exercise-associated cough in the absence of exercise-induced broncho-constriction (EIB). Two studies on children with asthma provided conflicting results regarding the relationship between FeNO and EIB. Terada et al. (8) stated that FeNO decreased during EIB, whereas Scollo et al. (9) concluded that FeNO did not change in children with asthma after an exercise challenge.

In the absence of any prospective study relating cough frequency to exercise and FeNO, we recruited 50 children with and without EIB. Our primary aim was to examine whether children with a pre-existing cough have an increase in cough frequency during and post-exercise and whether the presence of EIB and/or atopy influenced cough frequency. We also determined if any FeNO change post-exercise is related to the presence of atopy or EIB.

We hypothesized that children with any coughing illness have an increase in cough frequency post-exercise regardless of the presence of EIB or atopy. In addition, we hypothesized that FeNO levels decreases post-exercise regardless of the presence of EIB or atopy.

## Materials and Methods

### Participants

The inclusion criteria were children aged over 6 years. Coughers had a current chronic (duration >4 weeks) cough at the time of testing. Children with cough were recruited from the outpatient clinics when the clinicians looking after these children were querying if the cough was related to EIB. Controls were children without a current cough and were otherwise well. Controls were recruited from family and friends. We excluded children who were unable to either perform spirometry or run on a treadmill.

The study was approved by the Ethics Committee of the Children’s Health Services, Brisbane. Informed and written consent was obtained from all parents of participants and assent from appropriately aged children. The clinical trial was registered with the Australia New Zealand Clinical Trials Registry (ACTRN12607000511437).

### Protocol

**Figure 1:**
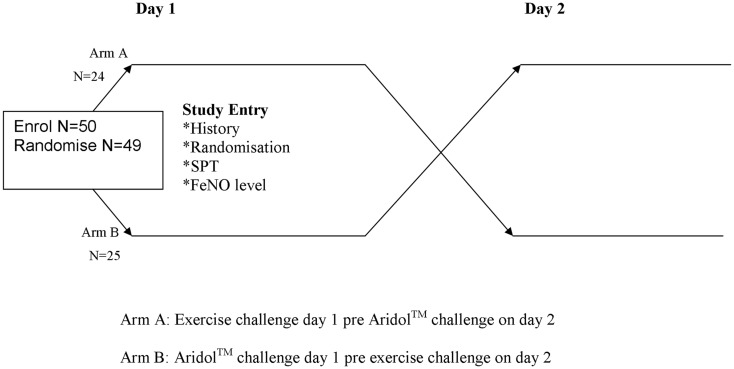
**Flowchart of methods**.

Children and their parents were approached when they attended a routine clinic visit at the tertiary hospital. After informed consent was obtained (Figure 1), we recorded the child’s demographics. A series of tests were performed over 2 days. On day 1 all children undertook: pre-baseline measurements (FeNO, spirometry); had a voice recorder fitted (for cough counts) and a skin prick test (SPT). Then the children were randomized to one of two sequences: (1) exercise challenge performed on day 1 with dry powder mannitol (Aridol™) challenge on day 2, or (2) mannitol challenge on day 1 with exercise challenge on day 2. Randomization was undertaken by an independent person off-site using a computer generation sequence list in permuted blocks 2–4. Allocation was fully concealed using opaque covers. Our primary outcome measures were cough frequency pre- and post-exercise challenge, and FeNO levels in children with cough and controls. In this manuscript, we are reporting data relating to the exercise test component of the study.

### FeNO, spirometry, and skin prick test

Fractional exhaled nitric oxide was measured with a chemiluminescence analyzer (Sievers NOA 280i, CO, USA) with children exhaling at 0.05 L/s for >4 s in order to obtain a stable nitric oxide value for >2 s, in accordance with ATS/ERS guidelines (10). Exhalations were repeated until three measurements were within 5% of the mean. Spirometry was performed after FeNO measurements using ATS/ERS criteria and the % predicted calculated based on height, age and sex matched reference values [Eigen (11) and Hibbert (12)].

Allergens used for SPT were alternaria mold, cat hair, cockroach mix, dust mite DPT, couch grass, and grass mix #7 (Hollister-Stier, WA, USA). Negative (diluents) and positive (histamine) controls were also used. Atopy was defined as wheal ≥3 mm larger than that of the histamine control after 15 min.

### Objective cough monitoring

A digital voice recorder (Sony PCP330F, Japan) recorded continuously for 24 h after being fitted post-randomization. The file (MP3) was downloaded and replayed. The time and number of coughs were manually counted by a person blinded to the child’s other data and recorded in a spreadsheet. Whilst the MP3 file was being listened to, the times of all challenges were recorded, enabling the coughs to be analyzed dependent on challenges. Additionally, the listener recorded the time the child seemed to be sleeping and awake. Wakefulness was defined as when the child could be heard talking or being active.

### Exercise challenge

The exercise challenge was undertaken in accordance to on ATS/ERS guidelines (13) using a treadmill (Trackmaster™ TMX425CP, USA). Room temperature and relative humidity were regulated by air conditioning to keep the water content ≤10 mg/mL (14). Children were required to withhold medications that could influence bronchial challenges (Table A1 in Appendix). Pulse oximetry was monitored during exercise testing. Treadmill slope and speed were adjusted to achieve target heart rates of at least 85% of maximum predicted (220 – age in years) within the first 2–4 min of exercise (15). Children continued exercise to achieve at least 4 min at the target heart rate, or, alternatively, until symptoms of dyspnea or fatigue required the patient to stop. Spirometry was performed post challenge at 1, 5, 10, and 15 min after the end of exercise. EIB was defined as a decrease in FEV_1_ ≥13% from baseline measurement (16).

### Statistical analyses

Descriptive statistics were used to summarize the demographic characteristics of the patients. Data that had a normal distribution were described using means and standard deviations (SD); medians and inter-quartile ranges (IQR) were used otherwise. Chi squared tests were employed for categorical data. Correlations were made using Spearman’s correlation (*r*_s_). Kruskal–Wallis analyses were used for group comparisons. Two-tailed *p* values of <0.05 were considered significant. All analyses were performed using a statistical package, SPSS Version 13.

## Results

We recruited 50 children between March 2006 and August 2008, of whom 49 were randomized. One child was not randomized as that parent did not consent to the mannitol challenge. One child (aged 6 years) commenced but could not complete the exercise challenge (cried) nor the mannitol challenge. Thus, data for cough and exercise were limited to 48 children (Table 1). Although the clinicians’ question was whether the current cough related to EIB, the underlying etiology of children with cough at the point of enrollment were: unknown, i.e., referred for determination of cough (*n* = 11, 33.3%), asthma (*n* = 10, 30.3%), cystic fibrosis (but referred for exercise test due to suspected EIB) (*n* = 8, 24.3%), suspected tracheomalacia (*n* = 1, 3%), congenital lobar emphysema (*n* = 1, 3%), follicular bronchiolitis (*n* = 1, 3%), and viral upper respiratory tract infection (*n* = 1, 3%). In the 11 children who did not have any diagnosis at the point of referral, the final diagnosis was asthma = 4, non-cystic fibrosis bronchiectasis = 2, and protracted bacterial bronchitis = 5.

**Table 1:** **Baseline data**.

	Coughers (*n* = 33)	Controls (*n* = 15)
**DEMOGRAPHICS**		
Boys:girls; *n* (%)	21 (63.6):12 (36.4)	5 (33.3):10 (66.7)
Age in years^a^	9.5 (7.8, 12.8)	11.75 (10.1, 14.8)
Atopy present; *n* (%)	16 (47.1)	3 (17.7)
**HISTORY**		
Prescribed asthma treatment; *n* (%)	20 (58.8)	
Duration of cough history (months)^a^	84 (48, 143)	N/A
**INVESTIGATIONS**		
FEV_1_ % predicted^a^	89.5 (82.6, 98.9)	95.0 (83.0, 99.0)
FeNO parts per billion^a^	12.1 (7.3, 34.0)	19.3 (11.5, 30.9)

*^a^ Median (IQR)*.

### Cough counts

**Figure 2:**
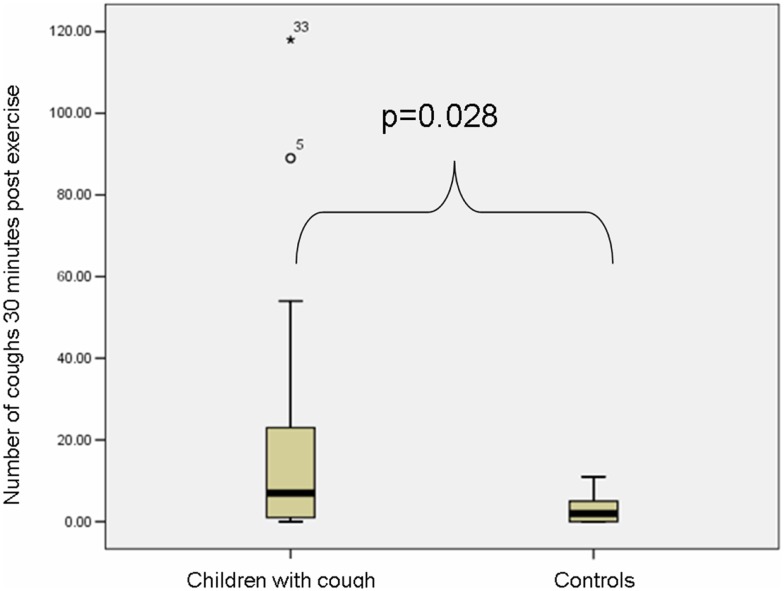
**Box plot (median and IQR) depicting the number of coughs 30 min post exercise in the coughers and controls**. The median number of coughs was significantly higher in the coughers compared to controls.

The median baseline cough count whilst the child was awake (total number of coughs not during airway challenges/hours awake) was significantly higher in the coughers compared to controls (Table 2). There was no significant difference between cougher and control groups in the number of coughs 30 min pre-exercise. However post-exercise, the median number of coughs was significantly higher in the coughers compared to controls (Figure 2). As the control children had little cough, the difference in cough frequency between 30 min pre and 30 min post-exercise was not significant between groups. None of the controls coughed during the exercise challenge but 13 of 33 children in the coughing group coughed [median number of coughs in these 13 children was 5 (IQR 2, 27)].

**Table 2:** **FeNO and objective cough counts between the groups**.

	Coughers (*N* = 33)	Controls (*N* = 15)	*p* Value
EIB (≥13% fall in FEV_1_); *n* (%)	7 (20)	0	0.06
FeNO at 5 min post exercise (ppb)^a^	9.2 (5.3, 28.1)	11.4 (7.2, 23.5)	0.37
Number of coughs in 30 min pre-exercise^a^	2.0 (0, 5.0)	0 (0, 2.75)	0.11
Number of cough in 30 min post-exercise^a^	7.0 (0.5, 24.5)	2.0 (0, 5.0)	**0.03**
Difference in cough counts (post 30 min minus pre 30 min)^a^	5.5 (0, 18.8)	0 (−2.2, 0)	0.09
Coughs/hr while awake (time excludes AHR challenges)^a^	6.7 (2, 12.2)	1.2 (0.4, 2.9)	**0.001**

*^a^ Median (IQR)*.

*The bold font highlights statistical significance*.

There was no significant difference in change of cough frequency in the 30 min post-exercise between children with and without EIB [EIB positive (*n* = 7): median 15.0 (IQR 0, 44.3); EIB negative (*n* = 31): median 5.0 (IQR 0, 16.0); *p* = 0.63]. There was also no difference in cough frequency between children grouped by presence of atopy [atopy present (*n* = 19): median 3.5 (IQR 1.7, 7.5); atopy absent (*n* = 29): 3.8 (IQR 0.7, 11.9); *p* = 0.91]. There was no difference in cough frequency post exercise when grouping by diagnosis in the children with cough (*p* = 0.78).

### FeNO levels

At baseline measurement of FeNO, there was no significant difference (*p* = 0.089) between the coughers and controls (Table 1). In both groups, FeNO levels fell post-exercise at 5 min compared to baseline and there was no significant difference between groups [Coughers: median −2.6 ppb (IQR −0.55, −4.35]; controls: −5.9 (IQR −1.0, −7.4), *p* = 0.158]. The fall in FeNO was larger in children with EIB [median −7.5 (IQR −3.0, −15.3)] compared to those without EIB (median −2.5, IQR −0.7, −5.98) but the difference did not reach significance (*p* = 0.06). There was no significant difference in decrease of FeNO between those with atopy (median −3.65, IQR −7.1, −0.88) and those without atopy (median −2.6, IQR −6.45, −0.75), *p* = 0.822.

## Discussion

To our knowledge, this study is the first to evaluate the effect of exercise on objective cough counts in children with and without a pre-existing cough. Our study found that with exercise, children with a current cough have an increase in cough frequency even in the absence of EIB or atopy. Also, the trend demonstrated a larger decrease in FeNO levels post-exercise in children with EIB, compared to those without EIB. Finally, atopy did not influence the change in FeNO levels post-exercise.

Cough with exercise or post-exercise is not rare and is sometimes used as a marker of asthma in children and adults (2, 3). Delineating whether exercise-associated cough really reflects asthma would be useful in clinical practice, particularly in children. Yet, to date there are no publications that have objectively quantified exercise-associated cough and examined its association with EIB in children. Objective quantification of cough is important in research studies on cough (5). In our cohort, albeit small, we found that post exercise cough objectively quantified was significantly higher in coughers (with and without EIB or atopy) compared to controls. This suggests cough frequency with exercise is a generic response and not limited to children with EIB. Whether or not those with EIB have an even greater increase in cough frequency post exercise compared to those without EIB remains unanswered as our sample size is too small.

In adults, several studies have examined cough in relation to several tests of airway hyper-responsiveness and have suggested that cough can be used as a marker of asthma. However, others have shown that medications used to abolish the broncho-constriction effect did not influence the cough response (17–19). In pediatric clinical practice, arguably delineating cough in relation to exercise is more applicable than that to other airway challenges. Suguikawa et al. evaluated cough in 20 adults with asthma who underwent methacholine, bradykinin, and exercise challenge tests (3). There was discordance among the tests for the frequency of cough. Bradykinin induced the most cough and exercise-induced the least (20). To our knowledge, the study by Suguikawa et al. (3) is the sole paper that have objectively monitored cough in relation to exercise.

As early as 1999, Rietveld and colleagues (21) questioned the use of cough alone as a marker of airway obstruction. In 30 children with asthma, they described the discordance between cough with other markers of asthma severity during a histamine challenge. Current Australian cough guidelines in children (22) recommend that chronic cough without any other features is seldom due to asthma, and inhaled corticosteroids are not indicated unless there are positive features to suggest asthma. However, the guidelines do not discuss cough with exertion and neither does the British Thoracic Society guidelines (23). Nevertheless, other, asthma-guidelines state that children with asthma may have an increase in cough during exercise (24), although this has never been evaluated using objective cough counts.

There are only a few studies that have related FeNO levels with cough or FeNO values post exercise. Our data showed a small but significant decrease in FeNO change post-exercise. Further, the fall in FeNO was small and within the coefficient of variant of the FeNO instrument used. Nevertheless, our data supports the findings of Terada et al. (8) but are in contrast to Scollo et al. (9) However, our study adds further novel data as neither studies examined FeNO in children with cough but without asthma. The fall in FeNO post exercise was larger in children with EIB and this difference between groups did not reach statistical significance. A larger study is required to examine this question. Several studies (25–27) conducted with healthy adults have found that during exercise FeNO decreases, whereas NO output increases.

As atopy is sometimes considered a surrogate of asthma in the presence of respiratory symptoms (20), we also examined the influence of atopy on our outcomes of interest. Our data showed that the presence of atopy did not influence the increase in cough frequency with exercise or 24-h cough frequency. This is consistent with our previous data showing that in children, cough sensitivity is not influenced by atopy (28).

Our study has many limitations that include a small sample size. A type-II error is thus invariably present for EIB and cough, as noted above. We had intended to recruit 100 children in total but the study was stopped early for feasibility reasons. Hence, in effect, this study represents pilot data. Accurate sample size calculation was not possible given the absence of data prior to this study. Secondly, we recruited children who were old enough to perform spirometry and exercise challenges and this limits applicability to younger children who are the largest age group of children referred for chronic cough (29). The studies were conducted more than 4 years ago but only recently analyzed. The delay related to personal circumstances of the primary author. We do not believe this represents a bias.

Another limitation is the heterogenous nature of the children with cough. However, our cohort represented real-life clinical settings where clinicians queried whether the current cough with exercise is a reflective of EIB and hence whether or not the child should receive more asthma medications. In clinical practice, even children with an underlying disorder such as cystic fibrosis may have increased cough with exercise that may or may not be reflective of their underlying respiratory diagnosis.

In spite of our study’s limitations, we have provided novel data relating cough frequency objectively measured with exercise in children with and without cough. Further we examined this relationship with respect to FeNO levels, EIB, and atopy. In conclusion, children with current cough have increased cough frequency post exercise even in the absence of EIB. In the absence of other symptoms, exercise-induced cough is likely a poor marker of asthma but a larger cohort study is required to verify whether children with EIB cough more than those without EIB in response to exercise.

## Conflict of Interest Statement

The authors declare that the research was conducted in the absence of any commercial or financial relationships that could be construed as a potential conflict of interest.

## Appendix

**Table A1:** **Drugs withheld prior to commencement of protocol**.

Long acting beta agonists	For 12 h
Beta-2-agonists	For 6–8 h
Singulair (leukotriene-receptor antagonist)	For 4 days
Sodium cromoglycate and nedocromil	For 8 h
Antihistamines	For 72 h
Inhaled corticosteroids	For 12 h
Caffeine (cola, chocolate, coffee, tea)	For 8 h
Strenuous exercise	For 6 h
